# Physiologic and pharmacokinetic changes in pregnancy

**DOI:** 10.3389/fphar.2014.00065

**Published:** 2014-04-03

**Authors:** Maged M. Costantine

**Affiliations:** Division of Maternal Fetal Medicine, Department of Obstetrics and Gynecology, University of Texas Medical BranchGalveston, TX, USA

**Keywords:** pregnancy, pharmacokinetics, pharmacology, physiology, fetus

## Abstract

Physiologic changes in pregnancy induce profound alterations to the pharmacokinetic properties of many medications. These changes affect distribution, absorption, metabolism, and excretion of drugs, and thus may impact their pharmacodynamic properties during pregnancy. Pregnant women undergo several adaptations in many organ systems. Some adaptations are secondary to hormonal changes in pregnancy, while others occur to support the gravid woman and her developing fetus. Some of the changes in maternal physiology during pregnancy include, for example, increased maternal fat and total body water, decreased plasma protein concentrations, especially albumin, increased maternal blood volume, cardiac output, and blood flow to the kidneys and uteroplacental unit, and decreased blood pressure. The maternal blood volume expansion occurs at a larger proportion than the increase in red blood cell mass, which results in physiologic anemia and hemodilution. Other physiologic changes include increased tidal volume, partially compensated respiratory alkalosis, delayed gastric emptying and gastrointestinal motility, and altered activity of hepatic drug metabolizing enzymes. Understating these changes and their profound impact on the pharmacokinetic properties of drugs in pregnancy is essential to optimize maternal and fetal health.

## INTRODUCTION

Prescription and over-the-counter medications use is common in pregnancy, with the average pregnant patient in the US and Canada using more than two drugs during the course of their pregnancy (Mitchell et al., 2001). One reason for this is that some women enter into pregnancy with pre-existing medical conditions, such as diabetes, hypertension, asthma, and others, that require pharmacotherapy; and for many others, gestational disorders (hyperemesis gravidarum, gestational diabetes, preterm labor) complicate women’s pregnancies and require treatment. Moreover, virtually the majority of organ systems are affected by substantial anatomic and physiologic changes during pregnancy, with many of these changes beginning in early gestation. Many of these alterations significantly affect the pharmacokinetic (absorption, distribution, metabolism, and elimination) and pharmacodynamic properties of different therapeutic agents (Pacheco et al., 2013). Therefore, it becomes essential for clinicians and pharmacologists to understand these pregnancy adaptations, in order to optimize pharmacotherapy in pregnancy, and limit maternal morbidity because of over- or under-treating pregnant women. The purpose of this review is to summarize some of the physiologic changes during pregnancy that may affect medication pharmacokinetics.

## CARDIOVASCULAR SYSTEM

**FIGURE 1:**
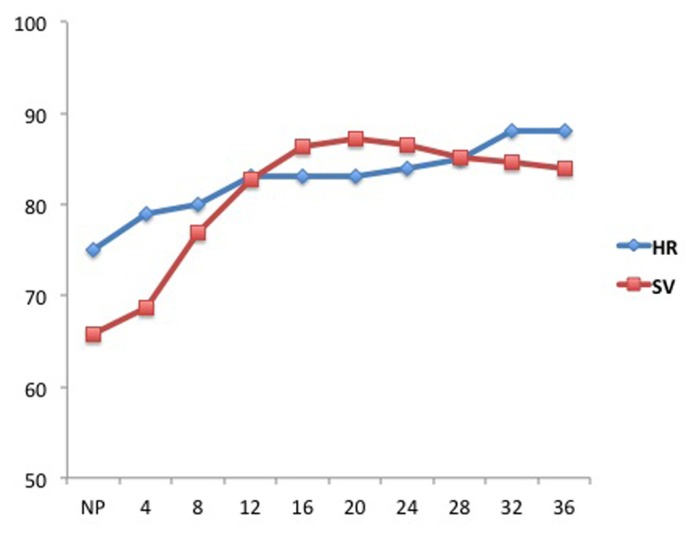
**Alterations in heart rate (HR, beats/min) and stroke volume (SV, mL) during pregnancy.** The X-axis represents gestational ages in weeks. NP represents the non-pregnant state (Figure adapted from Robson et al., 1989).

Pregnancy is associated with significant anatomic and physiologic remodeling of the cardiovascular system. Ventricular wall mass, myocardial contractility, and cardiac compliance increase (Rubler et al., 1977). Both heart rate and stroke volume increase in pregnancy leading to a 30–50% increase in maternal cardiac output (CO) from 4 to 6 l/min (**Figure 1**; Clark et al., 1989). These changes occur primarily early in pregnancy, and 75% of the increase will occur by the end of the first trimester (Capeless and Clapp, 1991; Pacheco et al., 2013). CO plateaus between 28 and 32 weeks gestation, and then does not change significantly until delivery (Robson et al., 1989). During the third trimester, the increase in heart rate becomes primarily responsible for maintaining the increase in CO (Pacheco et al., 2013). This increase in CO is preferential in which uterine blood flow increases 10-fold (17% of total CO compared with 2% prepregnancy) and renal blood flow increases 50%; whereas there is minimal alterations to liver and brain blood flow (Frederiksen, 2001). In addition, when compared with nulliparous women, multiparous women have higher CO (5.6 vs. 5.2 l/min), stroke volume (73.5 vs. 70.5 mL), and higher heart rate (Turan et al., 2008). During labor and immediately after delivery, CO increases as a result of increased blood volume (300–500 mL) with each uterine contraction, and then secondarily to “auto-transfusion” or the redirection of blood from the uteroplacental unit back to the maternal circulation after delivery (Pacheco et al., 2013). As CO increases, pregnant women experience a significant decrease in both systemic and pulmonary vascular resistances (Clark et al., 1989). Secondary to the vasodilatory effects of progesterone, nitric oxide and prostaglandins, systemic vascular resistances, and blood pressure decrease early in pregnancy, reaching their lowest point at 20–24 weeks, and leading to physiologic hypotension. Following this decrease, vascular resistances and secondarily blood pressure begin rising again, approaching the pre-pregnancy values by term (Clark et al., 1989; Seely and Ecker, 2011). This is especially important in patients with preexisting hypertension and who are on antihypertensive drugs (Pacheco et al., 2013; **Table 1**).

**Table 1:** Summary of cardiovascular changes during pregnancy.

Variable	Change
Cardiac output	Increased by 30–50%
Stroke volume	Increases to a maximum of 85 mL at 20 weeks of gestation
Heart rate	Increased (approaches 90–100 beats/minute at rest during the third trimester)
Systemic vascular resistances	Decrease 21% (nadir at 20–24 weeks)
Pulmonary vascular resistances	Decrease by 34%
Pulmonary capillary wedge pressure	No significant change
Colloid osmotic pressure	Decreased by 14%
Hemoglobin concentration	Decreased

Starting at 6-8 weeks of gestation and peaking at 32 weeks, maternal blood volume increases by 40–50% above non-pregnant volumes (Hytten and Paintin, 1963). This, coupled with drop in serum albumin concentration, leads to decreased serum colloid osmotic pressure and hemodilutional anemia. Because of the increased compliance of the right and left ventricles in pregnancy, the pulmonary occlusion and central venous pressures remain fixed (Bader et al., 1955). While exact origin of the increased blood volume is not fully understood, the mechanism may be through nitric oxide mediated vasodilatation and increased arginine vasopressin production and mineralocorticoid activity, with water and sodium retention, leading to hypervolemia (Winkel et al., 1980). The pregnancy induced hypervolemia is thought to provide survival advantage to the pregnant women, protecting her from hemodynamic instability with the blood loss at the time of delivery (Carbillon et al., 2000; Pacheco et al., 2013).

The increase in total body water, blood volume, and capillary hydrostatic pressure increase significantly the volume of distribution of hydrophilic substrates. Clinically, a larger volume of distribution could necessitate a higher initial and maintenance dose of hydrophilic drugs to obtain therapeutic plasma concentrations. Additionally, because of the decrease in serum albumin concentrations and other drug-binding proteins during pregnancy; drugs, that are highly protein bound, may display higher free levels due to decreased protein binding availability, and thus higher bioactivity. For example, if a drug is highly (99%) bound to albumin in non-pregnant patients, a small drop in protein binding to 98% in pregnancy translates into doubling of the drug’s active fraction in pregnancy. Digoxin, midazolam, and phenytoin are examples of medications primarily bound to albumin (Pacheco et al., 2013).

## RESPIRATORY SYSTEM

Due to the increase in estrogen concentrations in pregnancy, the respiratory system undergoes anatomic changes leading to increased vascularity and edema of the upper respiratory mucosa (Taylor, 1961). This may explain the increased prevalence of rhinitis and epistaxis during pregnancy. Although it is a theoretical risk and no studies have shown increased toxicity, inhaled medications, such as steroids used to treat asthma, may be more readily absorbed by pregnant patients (Pacheco et al., 2013).

Pregnancy is associated with increase in tidal volume by 30–50%, which starts early in the first trimester. While the respiratory rate is not different compared to non-pregnant state, minute ventilation (the product of respiratory rate and tidal volume) is significantly increased, similarly, by 30–50%. These changes are mainly driven by the increase in progesterone concentrations in pregnancy (Elkus and Popovich, 1992; McAuliffe et al., 2002). In addition, the diaphragm is pushed 4–5 cm upward due to the increased intra-abdominal pressure from the enlarging uterus and fluid third spacing. This leads to bibasilar alveolar collapse, basilar atelectasis, and decreased in both functional residual capacity and total lung capacity decrease by 10–20% (Baldwin et al., 1977; Tsai and De Leeuw, 1982). The decrease in functional residual capacity may predispose pregnant patient to hypoxemia during induction of general anesthesia. The vital capacity remains unchanged, as the decreased expiratory reserve volumes are accompanied with increased inspiratory reserve volumes (Baldwin et al., 1977; Pacheco et al., 2013).

When evaluating blood gases in pregnancy, it is important to note that the arterial partial pressure of oxygen (PaO2) is normally increased to 101–105 mmHg and that of carbon dioxide (PaCO2) decreased to 28–31 mmHg. These changes are mainly driven by the increase in minute ventilation described above. The drop of PaCO2 in the maternal circulation creates a gradient between the PaCO2 of the mother and fetus, which allows CO2 to diffuse freely from the fetus, through the placenta, and into the mother, where it can be eliminated through the maternal lungs (Pacheco et al., 2013). In addition, maternal arterial blood pH is slightly increased to 7.4–7.45 and consistent with mild respiratory alkalosis. This alkalosis is partially corrected by increased renal excretion of bicarbonate, leading to reduced serum bicarbonate level between 18 and 21 meq/L, and reduced buffering capacity (Elkus and Popovich, 1992; Pacheco et al., 2013). This partially compensated respiratory alkalosis slightly shifts the oxy-hemoglobin dissociation curve rightward, thereby favoring dissociation of oxygen and facilitating its transfer across the placenta, but it also may affect protein binding of some drugs (Tsai and De Leeuw, 1982).

## RENAL SYSTEM

The effects of progesterone and relaxin on smooth muscles are also seen in the urinary system leading to dilation of the urinary collecting system with consequent urinary stasis, predisposing pregnant women to urinary tract infections (Rasmussen and Nielse, 1988). This is more common on the right side secondary to dextrorotation of the pregnant uterus, and the right ovarian vein that crosses over the right ureter.

Both renal blood flow and glomerular filtration rate (GFR) increase by 50%, as early as 14 weeks of pregnancy (Davison and Dunlop, 1984). The mechanisms behind the increase in GFR are probably secondary to vasodilation of afferent and efferent arterioles. The increase in GFR leads to decreased serum creatinine concentrations, so that when serum creatinine concentration is above 0.8 mg/dL during pregnancy, it may indicate an underlying renal dysfunction (Pacheco et al., 2013) The increase in renal clearance can have significant increase (20–65%) in the elimination rates of renally cleared medications leading to shorter half-lives. For example, the clearance of lithium, which used to treat bipolar disorder, is doubled during the third trimester of pregnancy compared with the non-pregnant state, leading to sub-therapeutic drug concentrations (Schou et al., 1973; Pacheco et al., 2013). Other drugs that are eliminated by the kidneys include ampicillin, cefuroxime, cepharadine, cefazolin, piperacillin, atenolol, digoxin, and many others (Anderson, 2005).

The kidneys are also mainly involved in water and sodium osmoregulation. Vasodilatory prostaglandins, atrial natriuretic factor, and progesterone favor natriuresis; whereas aldosterone and estrogen favor sodium retention (Barron and Lindheimer, 1984). Although elevated GFR leads to additional sodium wasting, the higher level of aldosterone, which reabsorbs sodium in the distal nephron, offsets this wasting (Barron and Lindheimer, 1984). The resulting outcome is one of significant water and sodium retention during pregnancy, leading to cumulative retention of almost a gram of sodium, and a hefty increase in total body water by 6–8 l including up to 1.5 l in plasma volume and 3.5 l in the fetus, placenta, and amniotic fluid. This “dilutional effect” leads to mildly reduced serum sodium (concentration of 135–138 meq/L compared with 135–145 meq/L in non-pregnant women) as well as serum osmolarity (normal value in pregnancy ~280 mOsm/L compared with 286–289 mOsm/L in non-pregnant women; Schou et al., 1973). Another consequence of this volume expansion is reduced in peak serum concentrations (Cmax) of many hydrophilic drugs, particularly if the drug has a relatively small volume of distribution.

## GASTROINTESTINAL SYSTEM

In pregnancy, the rise in progesterone leads to delayed gastric emptying and prolonged small bowel transit time, by ~30–50%. Increased gastric pressure, caused by delayed emptying as well as compression from the gravid uterus, along with reduced resting muscle tone of the lower esophageal sphincter, sets the stage for gastro-esophageal reflux during pregnancy (Cappell and Garcia, 1998). In addition, these changes alter bioavailability parameters like Cmax and time to maximum concentration (Tmax) of orally administered medications (Parry et al., 1970). The decrease in Cmax and increase in Tmax are especially concerning for medications that are taken as a single dose, because a rapid onset of action is typically desired for these medications (Dawes and Chowienczyk, 2001).

Drug absorption is also decreased by nausea and vomiting early in pregnancy. This results in lower plasma drug concentrations. For this reason, patients with nausea and vomiting of pregnancy (NVP) are routinely advised to take their medications when nausea is minimal. Moreover, the increased prevalence of constipation and the use of opiate medications to ease pain during labor slow gastrointestinal motility, and delay small intestine drug absorption. This may lead to elevated plasma drug levels postpartum (Clements et al., 1978). The increase in gastric pH may increase ionization of weak acids, reducing their absorption. In addition, drug-drug interaction becomes important as antacids and iron may chelate co-administered drugs, which further decreases their already reduced absorption (Carter et al., 1981).

The increase in estrogen in pregnancy leads to increase in serum concentrations of cholesterol, ceruloplasmin, thyroid binding globulin, and cortisol binding globulin, fibrinogen and many other clotting factors (Lockitch, 1997). Serum alkaline phosphatase is elevated during pregnancy as it is also produced by the placenta, and its levels in pregnant women may be two to four times those of non-pregnant individuals; therefore limiting its clinical utility when liver function or enzymes are assayed (Lockitch, 1997; Pacheco et al., 2013). The rest of liver function tests such as serum transaminases (SGOT, SGPT), lactate dehydrogenase, bilirubin, and gamma-glutamyl transferase are not affected (Lockitch, 1997).

Drug metabolism is also altered in pregnancy in part secondary to elevated sex hormones and changes in drug metabolizing enzymes including those involved in phase I (reduction, oxidation, or hydrolysis) or phase II (glucuronidation, acetylation, methylation, and sulfation) metabolism (Evans and Relling, 1999). Cytochrome P450 (CYP450) represents a family of oxidative liver enzymes, and is a major route of drug metabolism for many drugs. For example, CYP3A4 exhibits a broad substrate specificity that includes nifedipine, carbamazepine, midazolam, and the anti-retroviral drugs saquinavir, indinavir, lopinavir, and ritonavir as well as many other drugs (Evans and Relling, 1999; Schwartz, 2003; Mattison and Zajicek, 2006). Because CYP3A4’s abundance and activity increase in pregnancy, the clearance of its substrates is also increased, requiring dose adjustment (Little, 1999). Examples of changes in phase II metabolism include increased activity of the conjugating enzyme uridine 5′-diphospho-glucuronosyltransferase (UGT) 1A4, which leads to increased oral clearance of lamotrigine, one of its substrates (de Haan et al., 2004; Pacheco et al., 2013).

## HEMATOLOGIC AND COAGULATION SYSTEMS

White (WBC) and red blood cell (RBC) counts increase during pregnancy. The first is thought to be secondary to bone marrow granulopoiesis; whereas the 30% increase in RBC mass (250–450 mL) is mainly driven by the increase in erythropoietin production. The higher WBC count can sometimes make diagnosis of infection challenging; however normally the increase in WBC is not associated with significant increase in bands or other immature WBC forms (Pacheco et al., 2013). Despite the increase in RBC mass, and as previously described, plasma volume increases significantly much higher (~45%), which leads to “physiologic anemia” of pregnancy. Anemia usually peaks early in the third trimester (30–32 weeks) and may become clinically significant in patients already anemic (iron deficiency, thalassemia, etc.) at entry to pregnancy (Pritchard, 1965; Peck and Arias, 1979). This physiologic hemodilution may provide survival advantage to women during pregnancy and childbirth, since the less viscous blood improves uterine and intervillous perfusion, while the increased red cell mass, coupled with increased uterine blood flow, optimizes oxygen transport to the fetus, and at the same time the blood lost during delivery will be more dilute (Koller, 1982; Letsky, 1995; Pacheco et al., 2013). The increase in RBC mass is accompanied by increased in maternal demand of iron by an additional 500 mg during pregnancy. This is coupled with an additional 300 mg of iron that is transferred to the fetus and 200 mg that is required for normal daily iron losses, making the total iron requirement in pregnancy around 1 g (Pacheco et al., 2013).

Pregnancy is a hypercoagulable state secondary to blood stasis as well as changes in the coagulation and fibrinolytic pathway such as increased plasma levels of clotting factors (VII,VIII,IX,X,XII), fibrinogen, and von Willebrand factor. Fibrinogen increases starting in the first trimester and peaks during the third trimester in anticipation of delivery. Prothrombin and factor V levels remain the same during pregnancy. Whereas, protein S decreases in pregnancy, protein C does not usually change and thus can be assayed if needed in pregnancy. Free antigen levels of the protein S above 30% in the second trimester and 24% in the third trimester are considered normal during pregnancy (Pacheco et al., 2013). Anti-thrombin III levels do not change, however, plasminogen activator levels are decreased and those of plasminogen activator inhibitor (PAI-1) levels increased by 2–3 fold, leading to suppressed fibrinolytic state in pregnancy. Platelet function and routine coagulation screen panels remain normal. This hypercoagulable state may offer a survival advantage by minimizing blood loss after delivery, but it also predisposes pregnant women to higher risks for thromboembolism (Hehhgren, 1996; Pacheco et al., 2013).

## ENDOCRINE SYSTEM

Plasma iodide concentration decreases in pregnancy because of fetal use and increase in maternal clearance of iodide. This predisposes the thyroid gland to increase in size and volume in almost 15% of women. In addition to anatomic changes, the thyroid gland increases production of thyroid hormones during pregnancy. This is due to the up-regulation of thyroid binding globulin, which is the major thyroid hormone binding protein, by almost 150% from a pre-pregnancy concentration of 15–16 mg/L to 30–40 mg/L in mid-gestation. This massive increase is driven by the hyper-estrogenic milieu in pregnancy and reduced hepatic clearance. The net result is increase in total tetra-iodothyronin and tri-iodothyronin hormones (TT4 and TT3) in pregnancy. Despite the increase in total T4 and T3, the free forms of the hormones (fT4 and fT3) remain relatively stable or slightly decreased but remain within normal values and these patients are clinically euthyroid (Glinoer, 1997; Glinoer, 1999; Pacheco et al., 2013). The increased thyroid hormones production takes place mostly in the first half of gestation, plateauing around 20 weeks until term. Clinically, due to these changes, the use of total T4, total T3 and resin tri-iodothyronine uptake is not recommended to monitor thyroid hormone status in pregnancy as they will be increased (TT4, TT3) and decreased (rT3U), respectively. For patients with hypothyroidism and who require levothyroxine replacement in pregnancy, it is recommended that they increase their levothyroxine dose by 30% early in pregnancy, be monitored during pregnancy, and to decrease the dose in the postpartum period (Alexander et al., 2004).

Thyroid stimulating hormone (TSH) decreases during the first half of pregnancy due to negative feedback from peripheral T3 and T4 secondary to thyroid gland stimulation by human chorionic gonadotropin (hCG). During the first half of pregnancy, a normal value of TSH is between 0.5–2.5 mIU/L (as compared to an upper limit of normal value for TSH of 5 mIU/L in the non-pregnant state). Other factors that affect thyroid hormones metabolism and levels in pregnancy include: (1) the increase in maternal renal iodine excretion (secondary to increase in GFR), (2) the higher maternal metabolic demands and rate during pregnancy, (3) the thyrotropic action of hCG which shares a similar α subunit with the TSH receptor and has a weak thyroid stimulating activity, (4) the increase in thyroid hormones transplacental transport to the fetus early in pregnancy, and (5) the increase in activity of placental type III 5-deiodinase (the enzymes that converts T4 to the inactive reverse T3; Glinoer, 1997; Glinoer, 1999; Pacheco et al., 2013).

## CONCLUSION

Profound physiologic and anatomic changes occur in virtually every organ system during pregnancy. These have significant consequences on the pharmacokinetic and pharmacodynamic properties of various medications when used by pregnant women. Data are lacking on the implications of these changes on variety of therapeutic agents, and future research is desperately needed.

## Conflict of Interest Statement

The author declares that the research was conducted in the absence of any commercial or financial relationships that could be construed as a potential conflict of interest.

## References

[B1] Alexander E. K. Marqusee E. Lawrence J. Jarolim P. Fischer G. A. Larsen P. R. (2004). Timing and magnitude of increases in levothyroxine requirements during pregnancy in women with hypothyroidism. *N. Engl. J. Med.* 351 241–249 10.1056/NEJMoa040079 15254282

[B2] Anderson G. D. (2005). Pregnancy-induced changes in pharmacokinetics: a mechanistic-based approach. *Clin. Pharmacokinet.* 44 989–1008 10.2165/00003088-200544100-00001 16176115

[B3] Bader R. A. Bader M. G. Rose D. J. Braunwald E. (1955). Hemodynamics at rest and during exercise in normal pregnancy as studied by cardiac catheterization. *J. Clin. Invest.* 34 1524–1536 10.1172/JCI103205 13263433 PMC438730

[B4] Baldwin G. R. Moorthi D. S. Whelton J. A. MacDonnell K. F. (1977). New lung functions in pregnancy. *Am. J. Obstet. Gynecol.* 127 235–239 835619 10.1016/0002-9378(77)90460-4

[B5] Barron W. M. Lindheimer M. D. (1984). Renal sodium and water handling in pregnancy. *Obstet. Gynecol. Annu.* 13 35–69 6371619

[B6] Capeless E. L. Clapp J. F. (1991). When do cardiovascular parameters return to their preconception values? *Am. J. Obstet. Gynecol.* 165 883–886 10.1016/0002-9378(91)90432-Q 1951547

[B7] Cappell M. Garcia A. (1998). Gastric and duodenal ulcers during pregnancy. *Gastroenterol. Clin. North Am.* 27 169–195 10.1016/S0889-8553(05)70352-6 9546089

[B8] Carbillon L. Uzan M. Uzan S. (2000). Pregnancy, vascular tone, and maternal hemodynamics: a crucial adaptation. *Obstet. Gynecol. Surv.* 55 574–581 10.1097/00006254-200009000-00023 10975484

[B9] Carter B. L. Garnett W. R. Pellock J. M. Stratton M. A. Howell J. R. (1981). Effect of antacids on phenytoin bioavailability. *Ther. Drug. Monit.* 3 333–340 10.1097/00007691-198104000-00003 7336470

[B10] Clark S. L. Cotton D. B. Lee W. Bishop C. Hill T. Southwick J. (1989). Central hemodynamic assessment of normal term pregnancy. *Am. J. Obstet. Gynecol.* 161 1439–1442 10.1016/0002-9378(89)90900-9 2603895

[B11] Clements J. A. Heading R. C. Nimmo W. S. Prescott L. F. (1978). Kinetics of acetaminophen absorption and gastric emptying in man. *Clin. Pharmacol. Ther*. 24 420–431 688732 10.1002/cpt1978244420

[B12] Davison J. M. Dunlop W. (1984). Changes in renal hemodynamics and tubular function induced by normal human pregnancy. *Semin. Nephrol.* 4 198

[B13] Dawes M. Chowienczyk P. J. (2001). Pharmacokinetics in pregnancy. *Best Pract. Res. Clin. Obstet. Gynaecol.* 15 819–826 10.1053/beog.2001.0231 11800526

[B14] de Haan G. Edelbroek P. Segers J. Engelsman M. Lindhout D. Devile-Notschaele M. (2004). Gestation-induced changes in lamotrigine pharmacokinetics: a monotherapy study. *Neurology* 63 571–573 10.1212/01.WNL.0000133213.10244.FD 15304599

[B15] Elkus R. Popovich J. (1992). Respiratory physiology in pregnancy. *Clin. Chest Med.* 13 555–565 10.1016/j.ccm.2010.11.001 1478018

[B16] Evans W. E. Relling M. V. (1999). Pharmacogenomics: translating functional genomics into rational therapeutics. *Science* 286 487–491 10.1126/science.286.5439.487 10521338

[B17] Frederiksen M. C. (2001). Physiologic changes in pregnancy and their effect on drug disposition. *Semin. Perinatol.* 25 120–123 10.1053/sper.2001.24565 11453606

[B18] Glinoer D. (1997). The regulation of thyroid function in pregnancy: pathways of endocrine adaptation from physiology to pathology. *Endocr. Rev.* 18 404–433 10.1210/edrv.18.3.0300 9183570

[B19] Glinoer D. (1999). What happens to the normal thyroid during pregnancy? *Thyroid* 9 631–635 10.1089/thy.1999.9.631 10447005

[B20] Hehhgren M. (1996). hemostasis during pregnancy and puerperium. *Hemostasis* 26 244–247 10.1159/000217305 8979130

[B21] Hytten F. E. Paintin D. B. (1963). Increase in plasma volume during normal pregnancy. *J. Obstet. Gynaecol. Br. Commonw.* 70 402–407 10.1111/j.1471-0528.1963.tb04922.x 13956023

[B22] Koller O. (1982). The clinical significance of hemodilution during pregnancy. *Obstet. Gynecol. Surv*. 37 649–652 10.1097/00006254-198211000-00001 7145246

[B23] Letsky E. A. (1995). Erythropoiesis in pregnancy. *J. Perinat. Med.* 23 39–45 10.1515/jpme.1995.23.1-2.39 7658318

[B24] Little B. B. (1999). Pharmacokinetics during pregnancy: evidence-based maternal dose formulation. *Obstet. Gynecol*. 93 858–868 10.1016/S0029-7844(98)00444-X 10912434

[B25] Lockitch G. (1997). Clinical biochemistry of pregnancy. *Crit. Rev. Clin. Lab. Sci.* 34 67–139 10.3109/10408369709038216 9055057

[B26] Mattison D. Zajicek A. (2006). Gaps in knowledge in treating pregnant women. *Gend. Med.* 3 169–182 10.1016/S1550-8579(06)80205-6 17081950

[B27] McAuliffe F. Kametas N. Costello J. Rafferty G. F. Greenough A. Nicolaides K. (2002). Respiratory function in singleton and twin pregnancy. *BJOG* 109 765–768 10.1111/j.1471-0528.2002.01515.x 12135212

[B28] Mitchell A. A. Hernandez-Diaz S. Louik C. Werler M. M. (2001). Medication use in pregnancy. *wPharmacoepidemiol. Drug Saf.* 10 S146

[B29] Pacheco L. Costantine M. M Hankins G. D. V. (2013). “Physiologic changes during pregnancy,” in *Clincal Pharmacology During Pregnancy* ed. Mattison D. R. (San Diego: Academic Press) 5–14

[B30] Parry E. Shields R. Turnbull A. C. (1970). Transit time in the small intestine in pregnancy. *J. Obstet. Gynaecol. Br. Commonw.* 77 900–901 10.1111/j.1471-0528.1970.tb03423.x 5473321

[B31] Peck T. M. Arias F. (1979). Hematologic changes associated with pregnancy. *Clin. Obstet. Gynecol.* 22 785–798 10.1097/00003081-197912000-00002 535196

[B32] Pritchard J. A. (1965). Changes in the blood volume during pregnancy and delivery. *Anesthesiology* 26 394–399 10.1097/00000542-196507000-00004 14313451

[B33] Rasmussen P. E. Nielse F. R. (1988). Hydronephrosis during pregnancy: a literature survey. *Eur. J. Obstet. Gynaecol. Reprod. Biol.* 27 249–259 10.1016/0028-2243(88)90130-X 3280355

[B34] Robson S. C. Hunter S. Boys R. J. Dunlop W. (1989). Serial study of factors influencing changes in cardiac output during human pregnancy. *Am. J. Physiol.* 256 H1060–H1065 2705548 10.1152/ajpheart.1989.256.4.H1060

[B35] Rubler S. Damani P. Pinto E. (1977). Cardiac size and performance during pregnancy estimated with echocardiography. *Am. J. Cardiol.* 49 534–540 10.1016/0002-9149(77)90068-6 910718

[B36] Schou M. Amdisen A. Steenstrup O. R. (1973). Lithium and pregnancy: hazards to women given lithium during pregnancy and delivery. *Br. Med. J.* 2 137–138 10.1136/bmj.2.5859.137 4699591 PMC1589270

[B37] Schwartz J. B. (2003). The influence of sex on pharmacokinetics. *Clin. Pharmacokinet.* 42 107–121 10.2165/00003088-200342020-00001 12537512

[B38] Seely E. W. Ecker J. (2011). Chronic hypertension in pregnancy. *N. Engl. J. Med.* 365 439–446 10.1056/NEJMcp0804872 21812673

[B39] Taylor M. (1961). An experimental study of the influence of the endocrine system on the nasal respiratory mucosa. *J. Laryngol. Otol.* 75 972–977 10.1017/S0022215100058746 13919984

[B40] Tsai C De Leeuw N. K. (1982). Changes in 2,3-diphosphoglycerate during pregnancy and puerperium in normal women and in beta-thalassemia heterozygous women. *Am. J. Obstet. Gynecol.* 142 520–523 7058853 10.1016/0002-9378(82)90754-2

[B41] Turan O. M. De Paco C. Kametas N. Khaw A. Nicolaides K. H. (2008). Effect of parity on maternal cardiac function during the first trimester of pregnancy. *Ultrasound Obstet. Gynecol.* 32 849–854 10.1002/uog.5354 18536067

[B42] Winkel C. A. Milewich L. Parker C. R. Jr. Grizzle W. E. Blevins J. K. Hawkes K. (1980). Conversion of plasma progesterone to desoxycorticosterone in men, non pregnant, and pregnant women, and adrenalectomized subjects. *J. Clin. Invest.* 66 803–812 10.1172/JCI109918 6968322 PMC371655

